# Hyper‐Intense Tumor Peripheral Accumulation of Antibody‐Conjugated Iron Oxide Nanoparticles can Enable Breast Cancer Detection by Magnetic Particle Imaging

**DOI:** 10.1002/advs.77425

**Published:** 2026-08-30

**Authors:** Preethi Korangath, Hayden Carlton, Theresa Wong, Sean Healy, Toby Sanders, Mason Song, Patrick W. Goodwill, Cordula Grüttner, Kathleen Gabrielson, Robert Ivkov

**Affiliations:** ^1^ Deptartment of Radiation Oncology and Molecular Radiation Sciences School of Medicine Johns Hopkins University Baltimore Maryland USA; ^2^ Magnetic Insight Inc. Alameda California USA; ^3^ Micromod Partikeltechnologie GmbH Rostock Germany; ^4^ Department of Molecular and Comparative Pathobiology School of Medicine Johns Hopkins University Baltimore Maryland USA; ^5^ Department of Mechanical Engineering Whiting School of Engineering Johns Hopkins University Baltimore Maryland USA; ^6^ Department of Oncology Sidney Kimmel Comprehensive Cancer Centre School of Medicine Johns Hopkins University Baltimore Maryland USA; ^7^ Department of Materials Science and Engineering Whiting School of Engineering Johns Hopkins University Baltimore Maryland USA; ^8^ Institute For NanoBioTechnology Whiting School of Engineering Johns Hopkins University Baltimore USA

**Keywords:** antibody‐conjugated nanoparticles, iron oxide nanoparticles, magnetic particle imaging, spatial analysis, tumor‐associated inflammation, tumor microenvironment, tumor targeting

## Abstract

Targeting nanoparticles to cancer cells in vivo remains a challenge for cancer imaging. We assessed nanoparticle distribution after injecting iron oxide nanoparticles conjugated with either a monoclonal anti‐HER2 (human epidermal growth factor 2), or a non‐specific IgG antibody into a human HER2 overexpressing murine breast cancer model. We used Magnetic Particle Imaging (MPI) and histopathology to assess particle localization at 72 h after injection. MPI detects magnetic moments produced by magnetic particles, and histology enables spatial quantification of nanoparticles. Intratumor iron content measured by MPI correlated with inductively coupled plasma mass spectrometry (*ρ* = 0.868, *p* < 0.0001). We detected nanoparticle accumulation in tumors, and organs and tissues associated with inflammation. MPI showed higher uptake of nanoparticles in tumors, regardless of their performance in vitro. Spatial analysis showed that 43 ± 7% of nanoparticles that reached the tumor accumulated in the peripheral quartile of the tumor, with decreasing amounts toward the center. Immunohistochemical analysis showed the nanoparticles were strongly associated with inflammatory immune and stromal cells in the tumor microenvironment. This hyperintense peripheral accumulation, or ring pattern, observed with MPI enabled us to distinguish tumors from general inflammation. Iron oxide nanoparticle‐mediated MPI has the potential to detect tumors, providing another powerful diagnostic tool.

## Graphical abstract

Tumor‐associated inflammation retains antibody‐targeted or non‐specific antibody conjugated nanoparticles within the tumor microenvironment. Magnetic Particle Imaging (MPI) enables non‐invasive detection and differentiation of tumors from other organs, owing to higher nanoparticle retention by inflammatory cells in the tumor periphery.

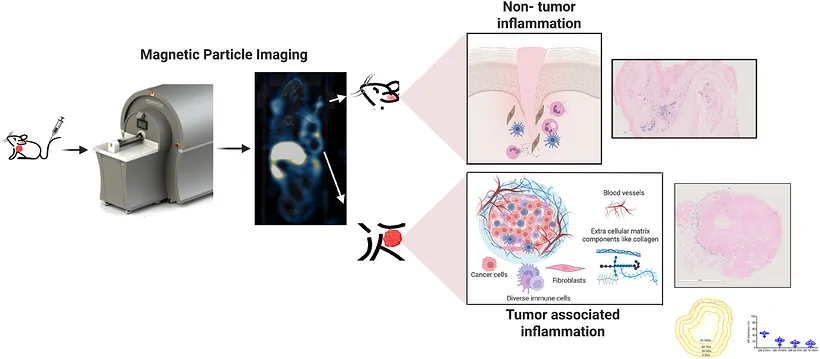

## 1 Introduction

Nanomedicine has advanced biomedical research over the past decade. Indeed, efforts such as the widespread implementation of liposomal mRNA vaccines during the COVID‑19 pandemic have led to significant clinical success of these agents [1]. It has been hoped that linking specific tumor‐targeting moieties, such as cancer‐specific antibodies to magnetic nanoparticles, will enable them to be directed to tumors and metastatic sites to aid both detection and treatment. Success in this endeavor would provide an opportunity to develop therapeutic antibody‐conjugated magnetic nanoparticles as theranostic agents. However, numerous challenges impede the clinical use of nanodrugs. Antibody‐conjugated nanoparticles enable robust immunohistochemical labeling of tumor‐specific receptors in vitro, but replicating these binding affinities in vivo remains a challenge. This is due, in part, to our evolutionarily programmed immune system, which recognizes, attacks, and sequesters/isolates the nanoparticles immediately to protect the host from foreign nanoparticles entering the body through the bloodstream [2]. These same cellular interactions between injected nanoparticles and host biology are also being recognized as a rational approach to designing nanoparticles for biomedical applications, since they can be exploited for delivery of therapeutic agents [3]. The underlying mechanisms of nanoparticle transport within solid tumors, however remain a source of debate [4, 5, 6, 7]. Conflicting evidence suggests intratumor nanoparticle retention is mediated either by passive (enhanced permeability) and/or active (receptor‐mediated) transport [8, 9]. New methodologies are needed to better understand the in vivo pharmacokinetics and biodistribution of nanoparticles.

Existing clinical imaging technologies, such as magnetic resonance imaging (MRI), sometimes use magnetic nanoparticles to aid detection of solid tumors. Improving existing modalities and developing new imaging techniques, such as magnetic particle imaging (MPI), are efforts intended to provide greater diagnostic capability for earlier (i.e., lower disease burden) and more accurate disease detection [10, 11, 12]. MPI is a newly developed “cold tracer” imaging technology that detects magnetic moment fluctuations of (ferro)magnetic or superparamagnetic materials when they are exposed to an alternating magnetic field [13, 14]. MPI signal intensity is directly proportional to nanoparticle concentration, and it can be used to detect nanogram quantities of magnetic materials in a living system [15]. Because the signal arising from the surrounding tissue is weakly diamagnetic, the background signal is negligible in MPI scans. Colloidal iron oxide nanoparticles (IONPs) have been used as parenteral agents for more than a century for iron deficiency anemia and other indications, and thus enjoy a proven safety profile in humans [16]. Their diverse biodistribution patterns depend very much on their chemical coating and physical characteristics, i.e. their physicochemical properties, which determine their biological performance [17, 18]. IONPs that reach the microenvironment of solid cancer tumors seem to be retained by various cells of the tumor microenvironment (TME), such as macrophages, neutrophils, and dendritic cells. These cells seem to sequester a significant amount of injected nanoparticles, regardless of the presence or type of ‘targeting’ moiety [19, 20]. Data show that nanoparticles, not bound by the liver, spleen, and other organs, tend to associate with immune and stromal cells in the TME, and rarely associate with the cancer cells to which they are presumably directed [19, 20]. Recently, we reported that a commercially available IONP formulation, pegylated Synomag, was retained in the microenvironments of tumors and metastases, thereby enabling non‐invasive detection of tumor tissue by MPI [15]. Microscopic analysis of IONPs retained in the TME provides further insights into these nanoparticle‐biology interactions. It is hoped these can be used to develop and improve nanoparticle‐based theranostic agents. In this study, we conjugated Synomag nanoparticles with a human HER2‐targeting monoclonal antibody or a non‐specific IgG antibody, and we evaluated their accumulation in the TME with MPI. We also investigated the nano‐bio interactions in the TME and other inflammatory regions by microscopic analysis to evaluate specific features that can be used to distinguish tumors from other inflammatory regions with this technique.

## 2 Results/Discussion

### 2.1 HER2 Antibody‐Conjugated Nanoparticles are Specifically Taken up by HER2^+^ Cells, In Vitro

**FIGURE 1:**
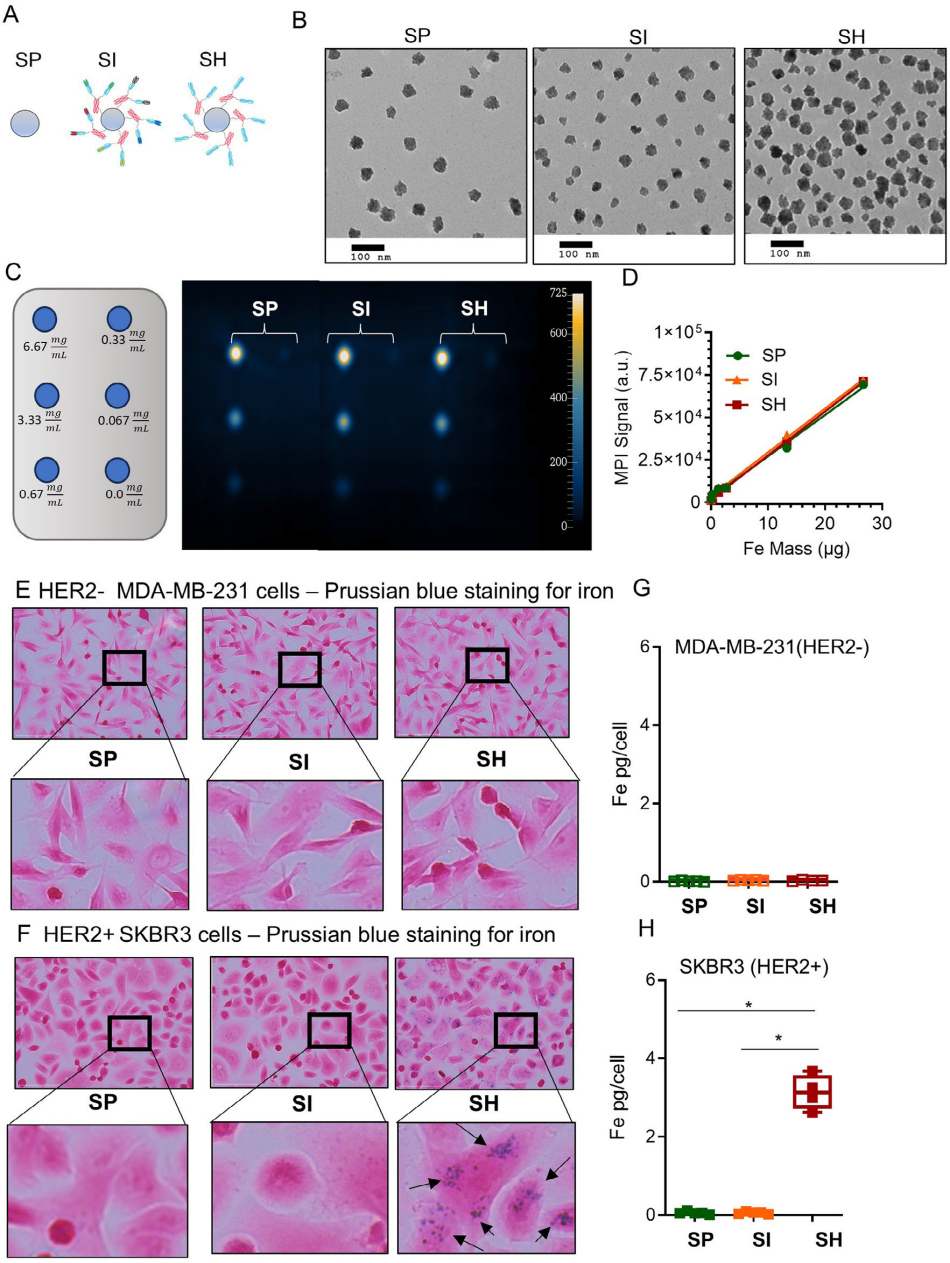
In vitro nanoparticle characterization. (A) Schematic of Synomag nanoparticles used in this study. A detailed protocol for conjugating non‐specific IgG and anti‐HER2 antibody is provided in the Methods. (B) Transmission electron microscope (TEM) images of unconjugated Synomag (SP), non‐specific IgG conjugated Synomag (SI), and anti‐HER2 antibody conjugated Synomag (SH) shows nanoflower core structure. (C) Two‐dimensional Magnetic particle imaging (MPI) scan showed a concentration‐dependent MPI signal without any difference among SP, SI, or SH nanoparticles at the same concentration. (D) A concentration‐dependent linear increase in the MPI signal corresponding to the iron concentration was noted for all three nanoparticles without any difference among the particles at any concentration. (E, F) Prussian blue staining visualizing iron oxide nanoparticles after 3 h incubation shows no internalization in HER2^−^ MDA‐MB‐231 cells, while a targeted uptake of SH nanoparticles is observed in HER2^+^ SKBR3 cells. (G, H) In vitro iron uptake Ferene assay demonstrated significant uptake of only SH nanoparticles in HER2^+^ SKBR3 cells and negligible uptake in HER2^−^ MDA‐MB‐231 cells after a 3 h incubation. None of the other nanoparticles were internalized by MDA‐MB‐231 or SKBR3 cells. The median and range of four independent experiments are shown. (Mann‐Whitney—*p < 0.05).

Synomag nanoparticles (SP) with conjugated antibody were used for the studies. SP are iron oxide nanoparticles with a nanoflower core and a hydroxyethyl starch coating (Figure 1A). The SP nanoparticles were conjugated either with a monoclonal anti‐HER2 antibody (SH), or a non‐specific, polyclonal, human IgG (SI) (See Methods). We compared the conjugated nanoparticles against the unconjugated parent SP. The hydrodynamic diameter of the SP nanoparticles was 106.7, the polyclonal antibody conjugated SI nanoparticles were 109.6 nm, and the SH nanoparticles were 127.4 nm. Measured zeta potentials were −0.25, +6.3, and −4.9 for SP, SI, and SH, respectively (Table S1). Transmission electron microscopy (TEM) images showed a flower‐shaped core structure (Figure 1B). We then characterized the linearity between the 2D MPI signal and nanoparticle mass for each nanoparticle type (Figure 1C). All three nanoparticle types displayed linear relationships between MPI signal and concentration, with a statistically insignificant difference in the slopes (Figure 1D) (*p* = 0.3). The similarity of the MPI signal with nanoparticle concentration for the three nanoparticle types enables direct comparison of the MPI signal from these nanoparticles without normalization. In vitro Prussian blue (Figure 1E,F) and ferene‐s assays (Figure 1G,H) were performed to test the specificity of the antigen/antibody‐mediated uptake of the SH nanoparticles in HER2^−^ MDA‐MB‐231 and HER2^+^ SKBR3 cells [19]. As expected, SH nanoparticles were specifically taken up by SKBR3 cells, with little uptake by the MDA‐MB‐231 cells, and neither cell line took up SP nanoparticles. This confirmed a targeted uptake of anti‐HER2 antibody‐coated SH nanoparticles by HER2^+^ (SKBR3) cells, in vitro. We also performed an in vitro ferene assay to rule out the possibility of protein corona formation that might hinder the specific uptake of HER2 antibody coated nanoparticles while they are in blood circulation. For this, we pre‐incubated the SH nanoparticles for 4 h in media with 10% serum at 37°C and then tested this with the ferene uptake assay as described in the methods. The results showed there was no significant difference in uptake of SH nanoparticles by HER^+^ SKBR3 cells with or without pre‐incubation (Figure S1A).

### 2.2 Biodistribution Analysis Showed Nanoparticle Accumulation in Various Organs and Tumors by MPI that Correlated With ICP‐MS

**FIGURE 2:**
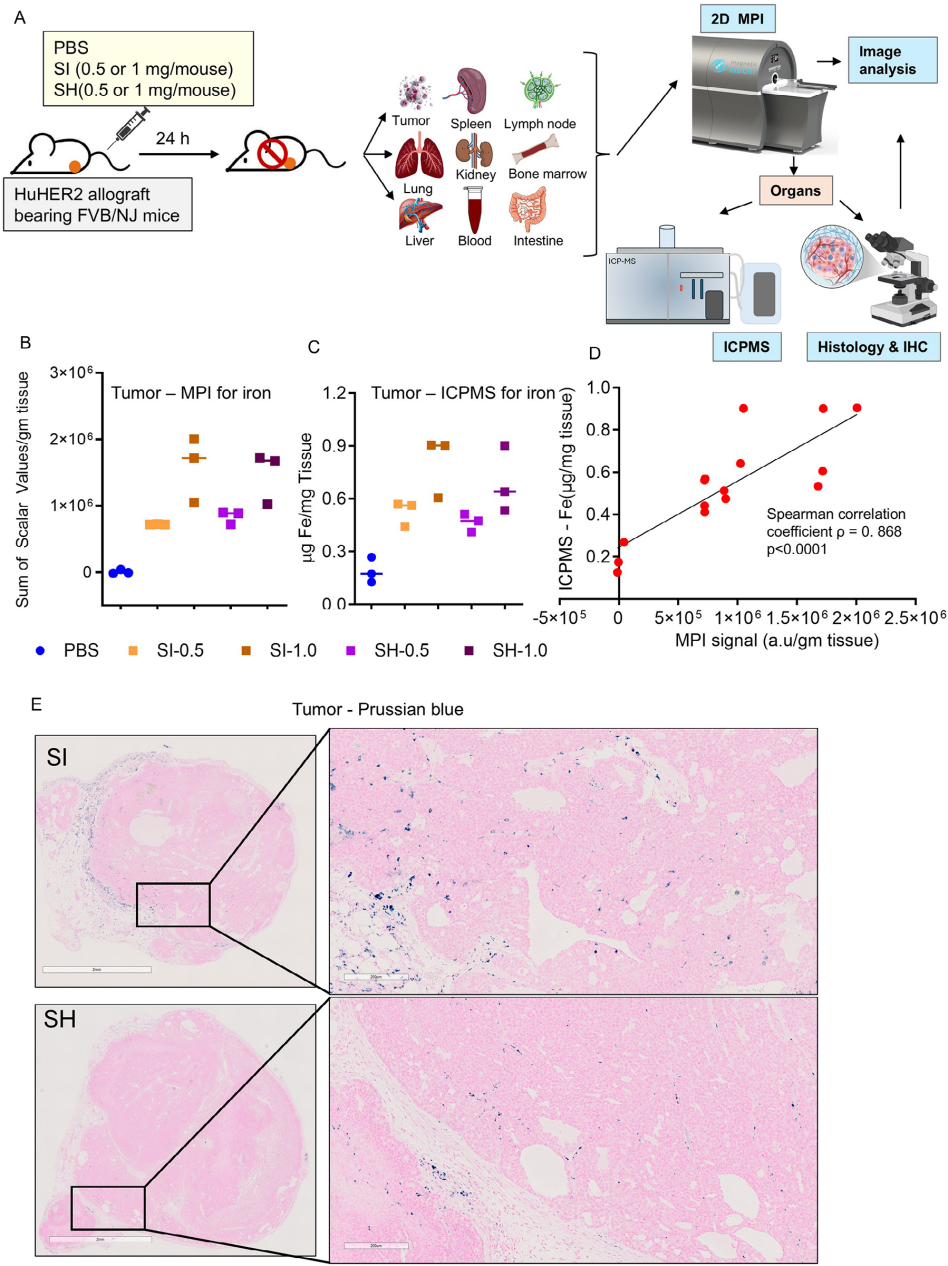
Biodistribution analysis by two‐dimensional MPI and ICPMS shows a positive correlation. A) Schema of study. Details in methods. (B) Dose‐dependent MPI signal was detected in tumors of mice injected with either SI or SH nanoparticles. No difference in uptake was observed between SI or SH at the same dose of injection, which indicates a general uptake rather than tumor antigen‐specific uptake. (C) ICPMS shows a similar trend of dose‐dependent nanoparticle accumulation in both SI and SH groups. (D) Tumor nanoparticle MPI signal correlated positively with measured iron by ICPMS (Spearman Correlation coefficient *ρ* = 0.868, *p* < 0.0001). (E) Histology analysis of the tumors after Prussian blue staining showed nanoparticle accumulation within the stromal regions of the tumor and the surrounding stroma.

We grew HuHER2 allograft tumors in FVB/NJ wildtype mice [19], injected nanoparticles intravenously into those mice when their tumors were 150–200 mm^3^ (total volume) and sacrificed them 24 h after injection to collect organs for quantitative analysis. Analysis included ex vivo MPI, inductively coupled plasma mass spectrometry (ICP‐MS), and histology (Figure 2A). Both SI and SH nanoparticles were injected at two different doses, 0.5 and 1.0 mg Fe. We note no statistically significant difference in iron content in tumors between the SI or SH nanoparticle groups. Both MPI (Figure 2B) and ICP‐MS (Figure 2C) showed a positive, dose‐dependent image signal and iron concentration, respectively, in all tumors, regardless of moiety (Spearman correlation ρ = 0.868, *p* < 0.0001) (Figure 2D). This indicates that MPI and ICP‐MS (gold standard) provided comparable nanoparticle detection, with similar amounts of injected iron being sequestered by tumor‐associated tissues. Similar dose‐dependent MPI (and ICP‐MS) signals were detected in other organs, including livers, spleens, kidneys, lungs, intestines, lymph nodes, bone marrow, and blood (Figure S1B,C). As with tumors, we noted no significant difference between SI and SH nanoparticles when they were given at the same doses. Iron concentration assessed by either MPI or ICP‐MS showed good correlation in all organs assessed (Figure S1D); however, the similarity between moieties (SI and SH) was noteworthy.

The similar in vivo behavior of the SI and SH particles was confirmed by histology. Prussian blue staining of tumors revealed the presence of either SI or SH nanoparticles in the TME (Figure 2E), depending on injection dose. Furthermore, both the SI and SH IONPs seemed to be similarly concentrated around the tumor edges, as we previously observed with different nanoparticles [20].

### 2.3 Spatial Analysis Revealed a Peripheral Hyperintensity Pattern of Nanoparticle Distribution Associated With Immune and Stromal Cells in the TME

**FIGURE 3:**
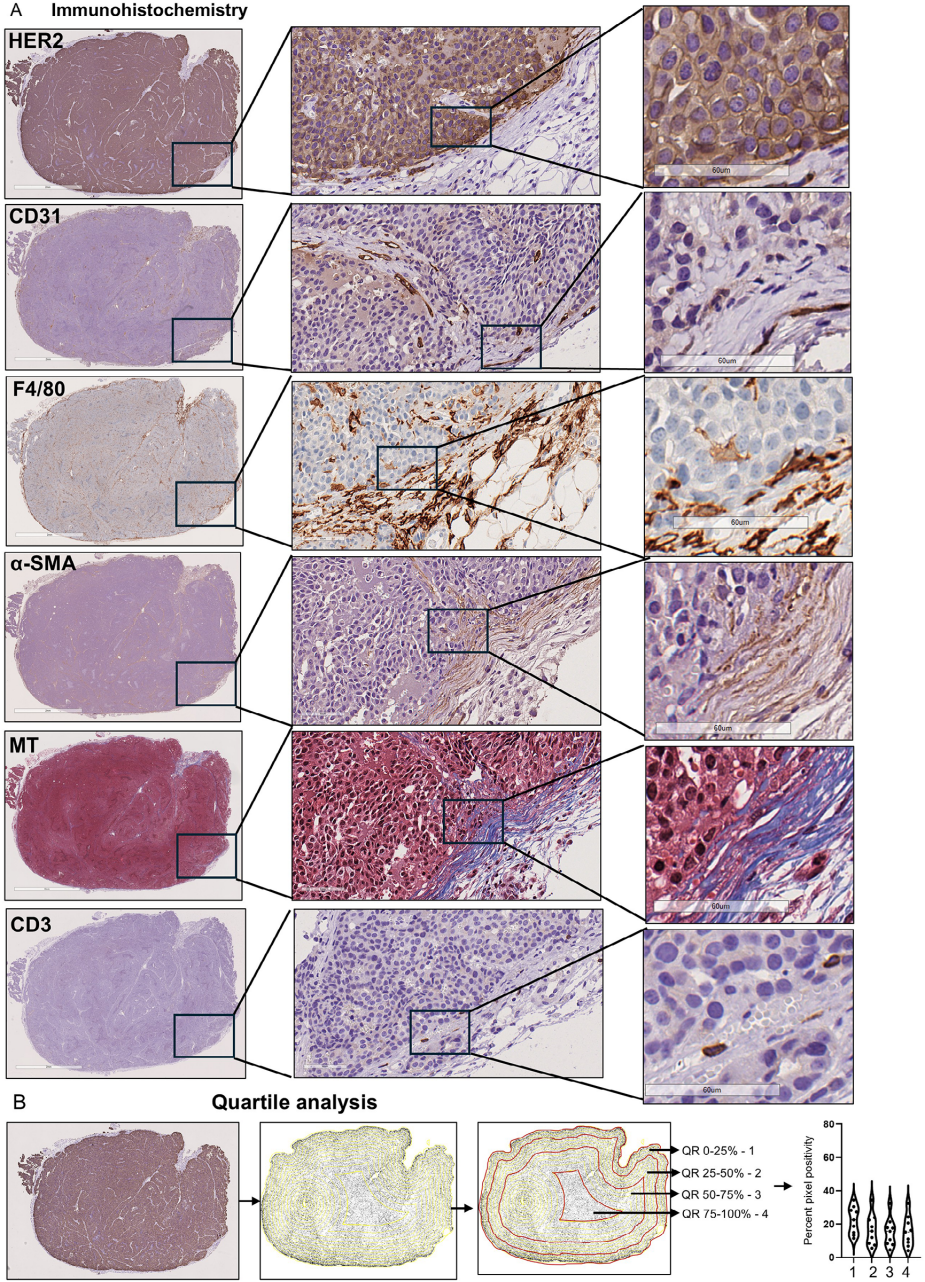
Spatial analysis of TME for immune and stromal cell distribution. (A) Histology and immunohistochemistry staining for various cell types. Tumor cells, endothelial cells, macrophages, fibroblasts, and T cells were identified by their markers HER2, CD31, F4/80, α‐SMA, and CD3, respectively. Masson's trichrome was used for collagen staining. (B) Representative image of tumor section stained for HER2 protein by IHC showing the workflow. Each stained slide was scanned and underwent image analysis. Each tumor was compartmentalized into quartiles of equal area from the edge toward the center. The pixel positivity representing cell type was quantified as described in the methods.

**FIGURE 4:**
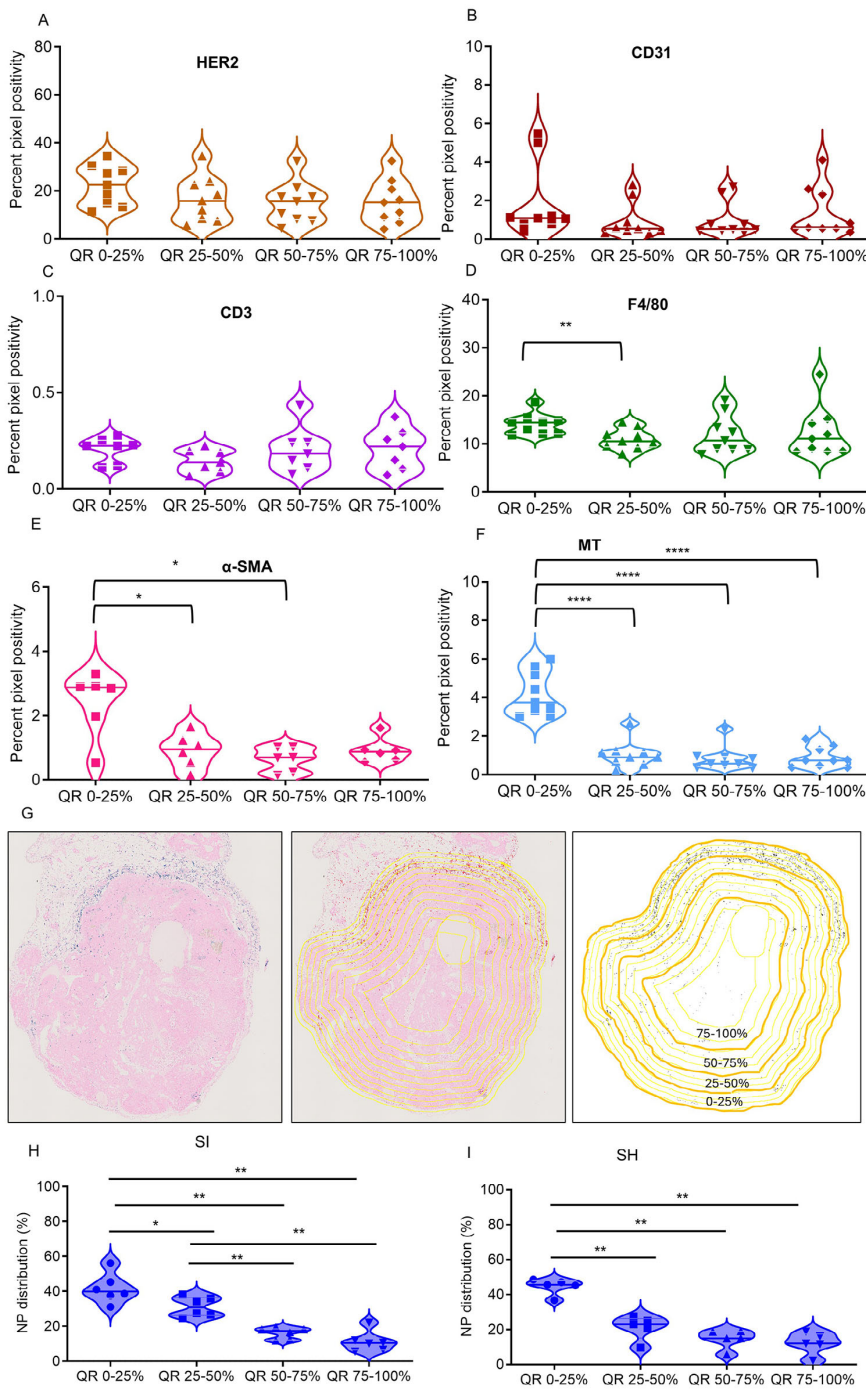
Quantitative analysis revealed a cell‐type‐specific pattern of distribution in TME that matched with nanoparticle distribution. (A–F) Spatial analysis of specific cells within each quartile showed that there was no significant difference in tumor cell (HER2+), endothelial cell (CD31+) or T cell distribution in the TME, while macrophages (F4/80+), fibroblasts (α‐SMA+), and collagen (Masson's trichrome+) showed significantly higher pixel positivity on the periphery of the tumor than inside. (G–I) Nanoparticle distribution in the tumor also showed peripheral accumulation, which was quantified as percentage distribution in each quartile of the tumor from edge to center. Significant accumulation in the outer quartile compared to the inner ones is noticed for both SI and SH cohorts, which follows the pattern of some immune and stromal cells in TME.

After performing intratumor iron quantitation using MPI and ICP‐MS, we performed spatial immunohistochemistry (IHC) analysis of different cell types in the HuHER2 allograft tumors. This analysis provided visualization and semi‐quantification for various cell types within the TME, which could then be compared with nanoparticle distribution. The cell types identified using their respective markers were tumor cells (HER2^+^), endothelial cells (CD31^+^), macrophages (F4/80^+^), fibroblasts (α‐SMA), collagen (Masson's trichrome), and T cells (CD3^+^) (Figure 3A). As described in prior work [20], we compartmentalized each tumor into four equal quartiles from the edge to the center and evaluated the number of positive pixels within each quartile to represent total cells in that area (Figure 3B). When we plotted the percent positive pixels for each cell type analyzed against the quartiles, we noted that tumor (Figure 4A), endothelial (Figure 4B), and T cells (Figure 4C) were distributed equally throughout the tumor, whereas macrophages (Figure 4D), fibroblasts (Figure 4E), and collagen (Figure 4F) were significantly higher toward the edges than the interiors of the tumors. We found a similar pattern of distribution for antibody‐conjugated nanoparticles in the TME of HuHER2 allografts, regardless of antibody type. Thus, we performed spatial quartile analysis of nanoparticle distributions obtained from Prussian blue staining in the TME (Figure 4G). As shown in Figure 4H, both SI (41 ± 8%) and SH (44 ± 4%) had approximately equal amounts of nanoparticles distributed on the edges, with the remainder distributed further inside the tumor, with amounts decreasing toward the tumor centers.

Intrigued by the findings, we performed a similar analysis on archived tumor tissue slides collected during a previous study. In that study, we injected a different iron oxide nanoparticle, Bionized nanoferrite (BNF), that was conjugated with IgG or HER2‐specific antibody [19]. The analysis was performed on HuHER2 allograft tumors in mice injected with PBS (control), unconjugated BNF plain (BP), non‐specific IgG conjugated BNF (BI) or anti‐HER2 antibody conjugated BNF (BH) 24 h after injection (Figure S2A). Quartile analysis was performed as described before (Figure S2B). As shown in Figure S2C, there was only baseline pixel positivity in tumors of PBS and injected mice, with no difference in their quartile density. BP had slightly higher pixel positivity than PBS, but showed no differences among quartile densities, most probably due to the low amount of nanoparticle accumulation in the mice injected with the bare BP (Figure S2D) compared to those injected with antibody‐conjugated nanoparticles [19]. But tumors from both groups injected with antibody‐conjugated BI and BH nanoparticles had significantly higher nanoparticle accumulation in their outer quartiles, which reduced toward the center. This peripheral hyperintense nanoparticle accumulation was akin to the patterns observed for macrophages, fibroblasts, and collagen distributions. Taken together, these data raise questions regarding our notions of nanoparticle ‘targeting’ to solid tumors, in vivo. Despite antibody‐specific uptake by cancer cells observed in vitro, the complex TME engulfs nanoparticles before they can reach the tumor cells in vivo. In other words, the TME and extracellular matrix trapping nanoparticles present a barrier to nanoparticle‐tumor cell interactions.

### 2.4 Nanoparticle Accumulation Coincides With Inflammation; MPI can be Used to Distinguish Between Primary Tumors and General Inflammation

We then tested a genetically engineered HuHER2 transgenic mouse model that produces spontaneous mammary tumors [21]. This spontaneous tumor model contrasts with the HuHER2 implanted tumor model, where the foreign cellular components, along with tumor cells during implantation, can lead to a heightened immune response from the host that may cause IONPs to get trapped in the TME, creating a false impression of immune cell involvement. The overall workflow is shown in Figure S3. We performed 3D MPI of tumor‐bearing HuHER2 transgenic mice after SP, SI or SH nanoparticle injection (1 mgFe). A total tumor burden of ∼1,500 mm^3^ was chosen as part of the inclusion criteria to ensure a higher probability of lung metastasis in these mice for imaging to evaluate the capability of MPI to detect lung metastases. A cohort of non‐tumor bearing age matched HuHER2 transgenic mice and wild‐type FVB/NJ mice served as controls. Following 3D imagery by MPI, all mice were subjected to µCT. All 3D MPI images shown here were reconstructed using an optimized physics‐based 3D reconstruction approach, shown previously, to preserve spatial fidelity, particularly in low‐sensitivity and high dynamic range scenarios [22, 23]. Many features agree with the default X‐space images from the scanner, but spatial resolution and interpretability were markedly improved with the optimized reconstruction. After 3D scans, mice were euthanized to collect individual organs of interest for 2D MPI scans. After scanning, all organs were then fixed for histology and immunohistochemistry (IHC) analysis.

**FIGURE 5:**
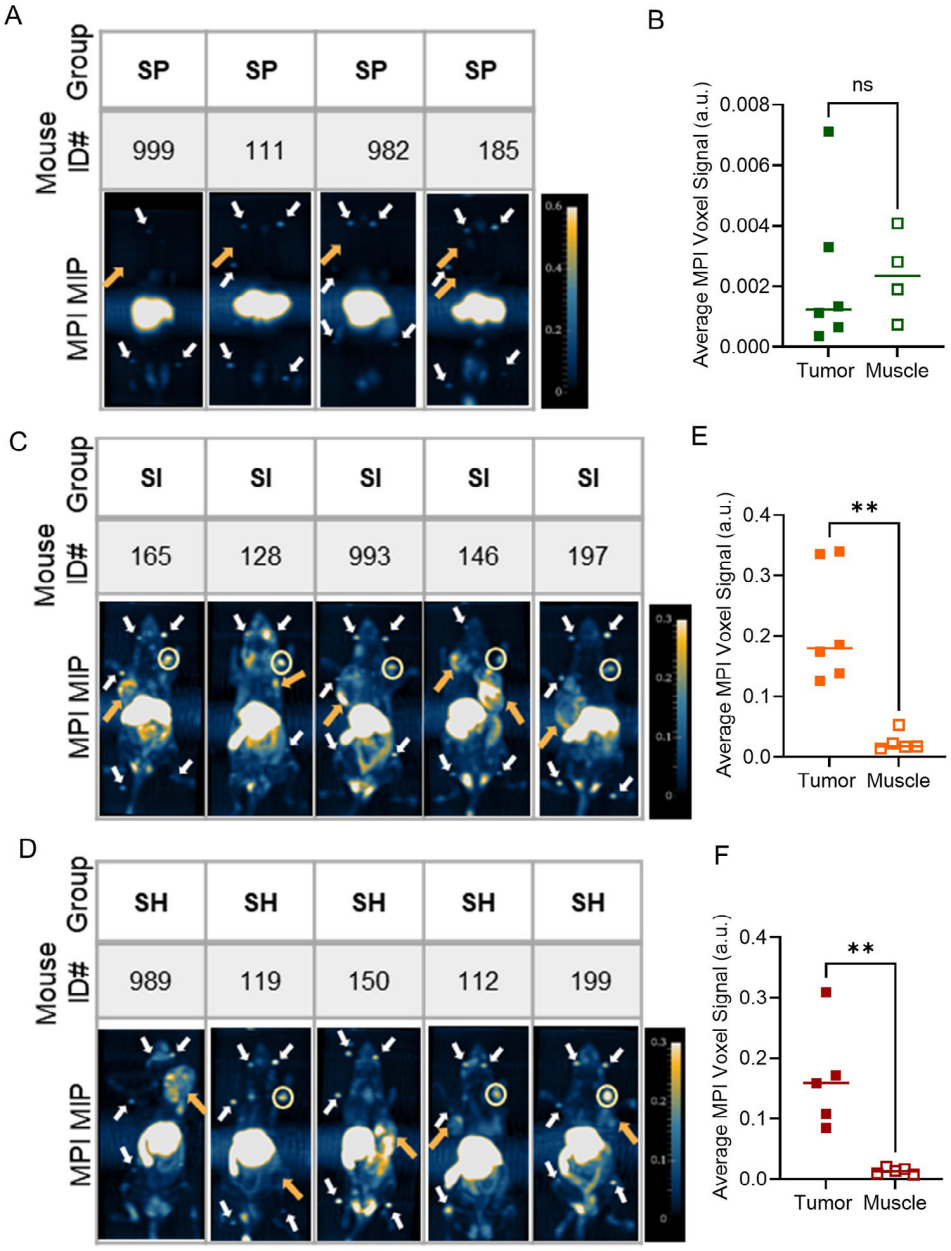
Surface coating‐dependent accumulation of nanoparticles on the tumor periphery enables their visualization by MPI. Maximum intensity projection (MIP) of whole body MPI scans shows selective retention of iron oxide nanoparticles in tumors. (A) Unconjugated SP injected into mice showed IONP signal only from the liver. No tumors were detectable (orange arrow). Fiducials are marked with white arrows. (B) Quantitative analysis of primary tumor area (35 mm^3^) with a similar area in the leg muscle region from the same mouse showed no significant signal difference. (C, D) Both SI and SH injected mice showed tumor accumulation apart from the liver, spleen, and lymph nodes. A high MPI signal was also noted in the right ear lobe area (yellow circle) of all SI and SH‐injected mice. The signal from tumors of SH nanoparticle‐injected mice was similar to that of SI cohorts. (E, F) Quantitative analysis of primary tumor area (35 mm^3^) with a similar area in the leg muscle region from the same mouse showed significantly higher MPI signal in both SI and SH cohorts. Graphs show all data points with the median. (**p < 0.01).

No significant differences were found among the treatment cohorts in terms of age, body weight, tumor volume or final tumor weight (Figure S4A–F). In the HuHER2 transgenic mice, we compared the 3D MPI scans from each group. The maximum intensity projections (MIPs) of mice injected with SP showed considerable nanoparticle signal in the liver, but virtually no signal was detected in the primary tumors (marked with orange arrows, Figure 5A). We quantitatively compared the 3D MPI signals from a small (∼35 mm^3^) region of the tumor with those from a similar volume of leg muscle. We observed no significant difference between tumor and muscle area in mice that received SP (Figure 5B). For the mice injected with either SI or SH, however, we noticed a substantially higher MPI signal in the tumors, enabling their visualization (Figure 5C,D).

There was a corresponding significantly lower signal in the liver than that observed in the SP group (Figure S4G). Conversely, quantitative analysis of the SI and SH groups showed significantly higher MPI signals in tumors than in the equivalent leg muscle area (Figure 5E,F). Additionally, we detected signal from major organs of the reticuloendothelial system (RES)—liver, spleen, and lymph nodes.

**FIGURE 6:**
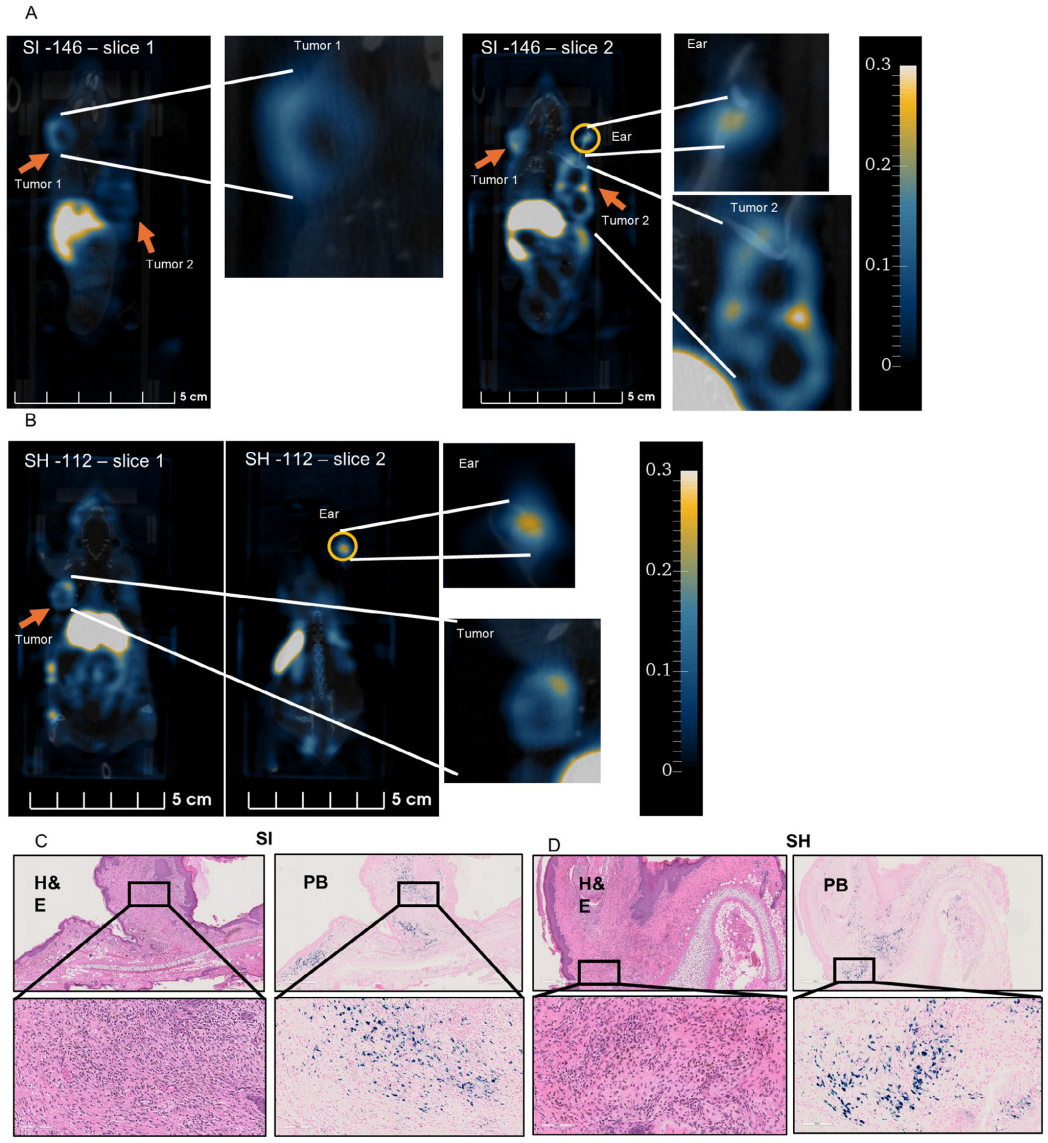
Hyperintense MPI signal around the tumor helps differentiate them from other inflammatory areas. (A, B) Representative images from SI and SH‐treated mice showing a hyperintense ring‐like structure around the tumors due to higher nanoparticle accumulation around the periphery (orange arrow). While the non‐specific right ear area was visually different from the tumor in all planes (yellow circle). Slices showing ring structures of the tumor at different planes of µCT co‐registered with MPI are shown. (C, D) Histological evaluation showed metal ear tag‐induced inflammatory changes in the right ear lobe of both SI‐ and SH‐injected mice. The area of inflammation showed nanoparticle retention by Prussian blue staining.

Multi‐planar reconstruction (MPR) of the MPI images showed a pattern of peripheral hyperintensity, showing as a ring of nanoparticle retention in the images (Figure 6A,B). This pattern was distinguishable from the bright spot of the non‐specific MPI signal seen in the right ear lobe (yellow circle) of SI and SH cohorts (Figure 5C,D and Figures S5 and S6), and the signal from liver and spleen, which appeared uniform throughout the image. Histological analysis of the ear tissue showed inflammatory infiltrates that engulfed nanoparticles (Figure 6C,D). The area where metal ear tags were clipped for identification was the area of inflammation. As reported previously, long‐term use of metal ear tags in small animals causes release of metal ions from the tags, leading to granulomatous inflammation and auricular chondritis [24, 25]. All mice with ear tags had some degree of inflammation near the area of the tag, but the MPI signal was detected only in those mice injected with antibody‐conjugated nanoparticles. The SP cohorts showed no MPI signal in the metal ear‐tagged lobe (Figure 5A). The presence of antibody‐conjugated nanoparticles in this inflammatory area, identified by MPI, further substantiates that inflammatory cells were responsible for the localization of the nanoparticles. It is possible that this occurs in addition to the activity occurring within tumor‐associated inflammatory cells.

This observation suggests that inflammatory cells or tissues, in general, preferentially sequester the antibody‐labelled nanoparticles, enabling their accumulation in specific areas. Nanoparticle accumulation at sites of inflammation would also include tumor and tumor‐associated tissues, which are often associated with inflammation. Nanoparticle sequestration at sites of inflammation is intriguing and should be studied further, as it may have a substantial influence on our ideas of nanoparticle uptake, perhaps even competing with the enhanced permeability and retention (EPR) mechanism thought to dominate in the tumor tissue itself. Although a high MPI signal was detected from the generalized inflammatory areas, differences in imaging patterns caused by the tumor‐specific peripheral uptake and its subsequent hyperintense pattern enabled us to distinguish the tumor from other tissues – i.e., generalized, non‐tumor inflammation and non‐specific organ uptake, e.g., liver and spleen, in MPI.

**FIGURE 7:**
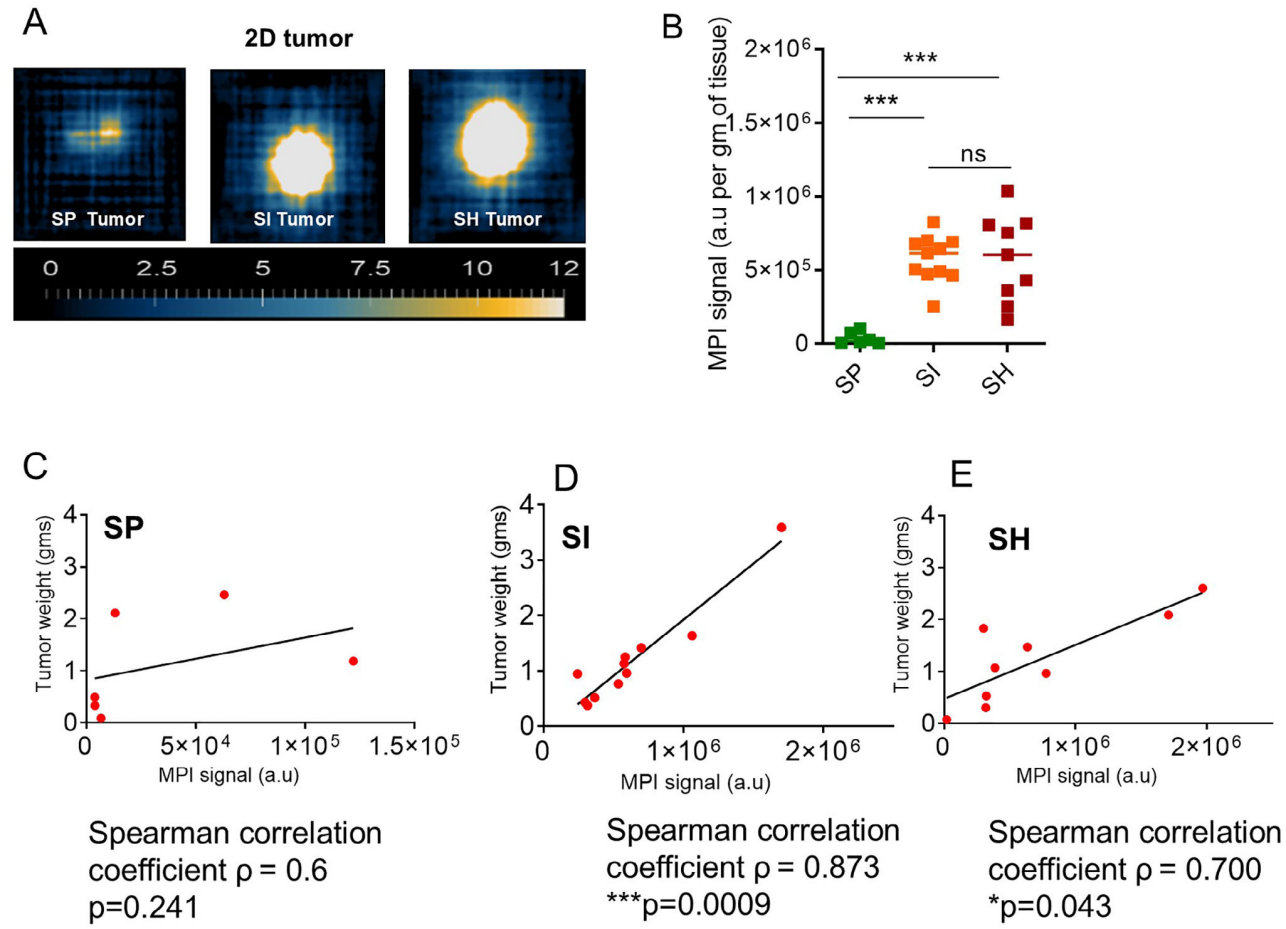
Two‐dimensional ex vivo MPI analysis of excised tumors showed a positive correlation of nanoparticles with tumor mass. (A) Representative images of ex vivo 2D MPI of tumors showing minimal positivity in the SP tumor and high signal in both SI and SH tumors. (B) Quantitative analysis revealed significantly higher MPI signal in SI and SH cohorts when compared to SP. The quantified MPI signal normalized to tumor weight is shown. (C–E) Correlation analysis showed a moderate to high positive correlation between the MPI signal and tumor mass in SP, SI, and SH cohorts. Note the scale difference in SP for MPI signal compared to SI and SH due to poor accumulation of SP nanoparticles in tumors. All data points as dots, with the median represented by lines. (*p < 0.05, **p < 0.01, ***p < 0.001).

The 2D ex vivo MPI scans of the excised tumors (Figure 7A,B) confirmed the observations in 3D, where mice injected with SP displayed minimal signal, while SI‐ and SH‐injected mice showed a relatively high signal intensity. The SI and SH groups showed significantly higher tumor MPI signal when compared with the SP cohort, but not when compared against each other. We may interpret this as a general retention of antibody‐coated nanoparticles in the tumors, regardless of specificity, but further study is warranted.

A positive correlation was observed between MPI signal and tumor weight in all cohorts injected with nanoparticles (Figure 7C–E), with SI and SH showing the highest Spearman correlation coefficient (*ρ* = 0.873 and 0.7, respectively). This indicates a higher number of inflammatory stromal and immune cells are present in the TME as the tumors grow [26, 27], and those cells are indiscriminately taking up nanoparticles with greater affinity toward antibody‐conjugated nanoparticles.

It has to be noted that the ex vivo MPI scans are 2D planar projections of the MPI signal obtained from excised tissues/organs, whereas the in vivo scans are full 3D scans of the intact mouse. While the 2D scans preserve the total MPI signal, information about nanoparticle distribution in the *z*‐axis (i.e., depth of peripheral accumulation) is lost. Hence, the 2D images do not show the peripheral accumulation pattern, while a 3D in vivo scan shows the full volumetric tomography of nanoparticle distribution, which allows us to see the peripheral distribution in 3D space. Additionally, a higher magnetic field (10 mT) was used for the ex vivo scans to enable detection of minute amounts of nanoparticles in individual organs and compare against parameters such as tumor weight. For in vivo whole‐body MPI, the magnetic field was set to 5 mT to mitigate saturating signals coming from regions with higher nanoparticle‐accumulating, such as the liver and spleen. Finally, post‐imaging analysis and reconstruction methods for 2D and 3D are different as described in the methods.

**FIGURE 8:**
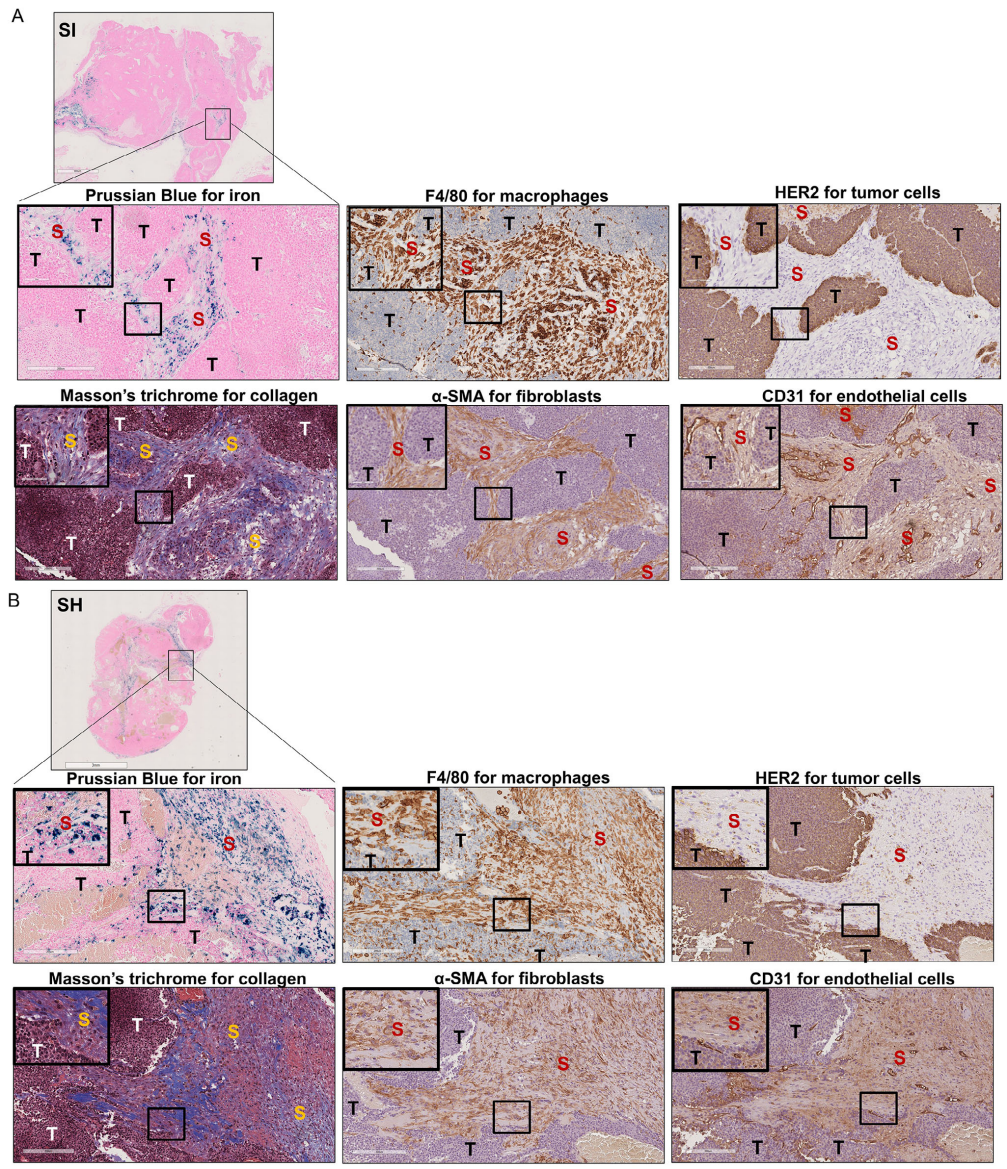
Nanoparticles are retained by tumor‐associated stromal and immune cells irrespective of antibody type conjugated to nanoparticles in HuHER2 transgenic mice. (A, B) Prussian blue staining for iron in tumor slices from SI‐ and SH‐injected mice show nanoparticle accumulation, predominantly in stromal areas surrounding and within the tumor. Immunohistochemistry for macrophages (F4/80), fibroblasts (α‐SMA), collagen (Masson's trichrome), and endothelial cells (CD31) showed positivity in those stromal areas where nanoparticles were observed by Prussian blue staining, whereas tumor cells identified by HER2 staining showed no substantial Prussian blue staining for IONPs. Taken together, these results indicated a predominant association of nanoparticles with stromal and immune cells, but not with tumor cells. Tumor regions are marked as T and stromal regions as S.

After performing quantitative MPI, we analyzed the TME of the HuHER2 transgenic mice using IHC, as described above. This provided microscopic visualization of a single portion of a 5 µm‐thick section of tumor tissue in the 2D plane. As with the allograft mouse models, both SI and SH tumors showed nanoparticles congregated around the tumor periphery and in the stromal areas within the tumor that also showed positive staining for macrophages, collagen, and fibroblasts but negative staining for tumor cells (Figure 8A,B). Pathologically, HuHER2 transgenic tumors resemble advanced human breast tumors, where clusters of tumor islands are seen with intertwining stromal components. Nanoparticles were observed throughout the stromal‐rich regions of the TMEs. Nanoparticle presence was noted near endothelial cells, indicating that the nanoparticles gained access to the tumor via the bloodstream. They may have then extravasated via endothelial cells as recently reported by Wu et al. [9], whereupon they were engulfed by stromal and immune cells, especially macrophages in the TME [28]. We found no detectable MPI signal in the blood (Figure S4G), confirming IONP clearance from the bloodstream, and its subsequent accumulation into various tissues, including tumors, within the time of measurement (72 h). Analysis of organs by MPI showed a significant accumulation of nanoparticles (Figure S4G,H) that corroborates the Prussian blue staining of those organs (Figure S7).

### 2.5 Nanoparticles were Associated With Inflammatory Cells in Micrometastatic Sites

**FIGURE 9:**
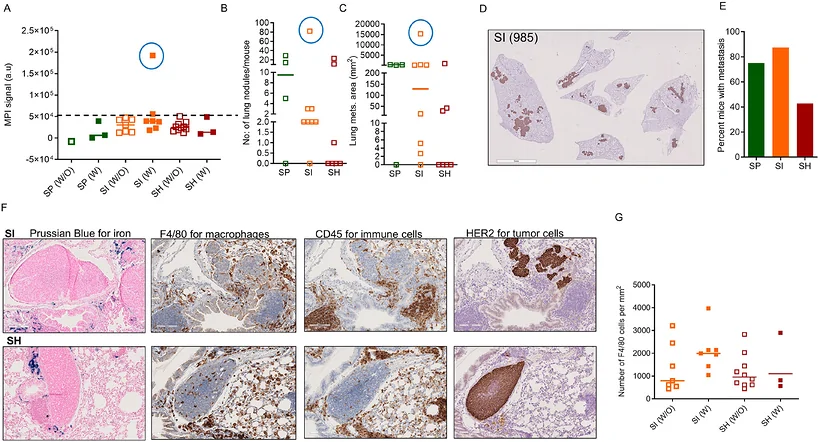
Ex vivo MPI from lungs of IONP‐injected mice showed no difference with and without micrometastases, while nanoparticles were seen retained in metastasis‐associated macrophages. (A) Quantitative 2D MPI from the lungs of mice injected with SP, SI or SH injected with (W) and without (W/O) micrometastases did not show any significant difference. (B, C) A statistically insignificant decrease in the number and the area of metastatic nodules was noted in SH‐treated mice compared to SP and SI groups. This may be indicative of some residual pharmacologic activity by the antibody. One outlier (circled in blue) in SI cohort had high MPI signal had a higher number and area of metastatic nodules in the lungs. (D) HER2 IHC of the lungs from that mouse is shown. (E) A reduced number of mice with lung metastasis was noted in SH injected cohort compared to SP and SI. (F) Histology analysis of lungs showed the presence of Prussian blue positive SI and SH nanoparticles in the tumor microenvironment. These, however, were confined to areas associated with immune cells (CD45+ regions), mostly with F4/80 macrophages surrounding micrometastatic nodules. They did not seem to associate with HER2^+^ tumor cells. (G) Quantitative analysis of F4/80 cells in lungs showed no significant difference between lungs with and without metastasis after SI or SH injection.

Some HuHER2 transgenic mice develop micrometastases in the lungs, which can only be confirmed by postmortem HER2 IHC of lung tissue. Since this model does not show a very high number or large metastatic nodules, we did not expect detection by 3D imaging. Also, the high signal from the liver obscures any distinguishable signal from the lungs due to high intensity ‘washout’. Hence, we analyzed individual organs (Figure S4G,H) from non‐tumor and tumor bearing mice, both injected with IONPs. We observed the total sum of liver signal to be about ∼10^6^ a.u., while the signal from the lungs was about ∼10^4^ a.u. (Figure 9, Figure S4). Even in a theoretical ideal scan, the degree of separation needed between the high and low signals for this degree of dynamic range is approximately 0.5 cm [15]. Further studies are ongoing to improve scanning parameters and data analysis algorithms to resolve this issue. Nevertheless, we performed additional ex vivo analysis of the lungs to better understand nanoparticle localization and MPI signals.

Two‐dimensional ex vivo lung scans of mice that received IONPs did not show differences in MPI signals among SP, SI or SH particle types. We also noted no statistically significant differences between lungs with or without HER2^+^ micro metastases (confirmed by histology) (Figure 9A). In our previous study using the same model with PEG‐Synomag, we reported a cutoff value of 50 346 a.u. for MPI‐based detection of metastases in lungs [15]. Only one mouse in this study, injected with SI, showed an MPI signal of 192 374 a.u. (marked with a blue circle), which was well above the cutoff value. This outlier was in the SI group and had both a higher number and a larger total area of HER^+^ metastatic nodules in the lungs (Figure 9B,C). We conclude that the higher MPI signal probably correlated with a higher nanoparticle accumulation in the larger tumor burden identified by HER2 IHC (Figure 9D).

Although statistically insignificant due to small sample size, we noticed a relatively higher number of primary tumor‐bearing mice having metastases in the SP (75.0%) and SI (87.5%) cohorts compared to the SH (42.8%) cohort (Figure 9E). We also noticed comparatively fewer nodules that were smaller in size in the SH cohorts than in the other two cohorts (Figure 9B,C). This tantalizing observation may indicate some residual therapeutic activity of the Herceptin antibody, whether free (i.e., antibody that was unbound after chemistry) or bound to nanoparticles. This observation warrants further investigation.

We then analyzed the location and association of SI and SH nanoparticles in the lungs by tissue histology. The region of tissue containing micrometastases showed decreasing tumor sections with each successive 5 µm section/cut, as the successive sections moved away from the micrometastatic nodule(s). This had the effect that images/slides appeared different at low magnification, even though they were images of the same slide (Figures S8 and S9). Prussian blue‐positive areas tended to be associated with the HER2^+^ micrometastases, indicating deposition of iron nanoparticles in those areas. We stained serial sections of these slides for both total immune cell populations and specifically macrophages with CD45 and F4/80 antibodies, respectively. We observed from this examination that nanoparticles were associated with immune cells in stromal areas of metastatic sites, but especially with the macrophages in those areas. While IONPs were observed to be localized around micrometastatic nodules, as previously seen with primary tumors, the localization was predominantly in areas with higher immune cell numbers than in primary HER2^+^ tumors, in both SI‐ and SH‐injected mice (Figure 9F, Figures S8 and S9). This qualitative assessment of lung tissue needs further evaluation in future studies with more mice for quantitative validation. We found a non‐significant increase in the number of macrophages in lungs bearing metastases in the SI group, but not in the SH group (Figure 9G). This suggests involvement of other cell types and even macrophage phenotype‐specific retention of nanoparticles in the TME.

In general, macrophage phenotypes change dynamically (space and time) in the TME based on their immediate microenvironmental signaling by cytokines [29, 30]. We assessed whether nanoparticle uptake depends on the phenotype of macrophages in vitro with immortalized RAW264.7 cells. As seen from Figure S10, macrophages induced to their proinflammatory phenotype with LPS and IFN‐γ engulfed significantly more nanoparticles than either naïve or anti‐inflammatory, M2, macrophages, irrespective of antibody conjugation.

Despite decades of investigation, targeting nanoparticles with an antigen‐specific antibody for both imaging and therapy remains elusive [31]. Our study sheds light on possible causes. Nanoparticles are often recognized and treated as foreign entities by the (mammalian) host immune system, which often exerts a profound effect on their uptake and retention. Furthermore, the immune status of an individual will have a variable and profound effect on nanoparticle performance, within parameters defined by species norms. Livers and spleens are typically macrophage‐abundant tissues, followed by the intestines, bone marrows and kidneys [32]. We observed a higher MPI signal (thereby higher nanoparticle retention) in these organs. By marking individual mice with metal ear tags, we inadvertently induced tissue inflammation in the ear lobes of the mice. Those areas showed increased retention of antibody‐labeled nanoparticles, both by MPI and histology. In the tumor, however, the specific pattern of higher nanoparticle accumulation was near tumor edges, suggesting retention by phagocytic inflammatory cells on the tumor‐normal tissue boundary. Both SI and SH nanoparticles showed similar patterns of distribution in the TME and the inflammatory ear lobes, indicating there was relatively little or no recognition of specific antigenicity. Rather, there was a greater dependence on the uptake and retention of nanoparticles depending on the coating. In other words, we showed that tumor‐associated inflammation was responsible for preferential antibody‐conjugated nanoparticle retention in TME, regardless of the antibody type.

The presence of antibody, especially human IgG, makes nanoparticles more conspicuous to host immune and stromal cells than non‐modified IONPs [33]. It is reported that the blood circulation time of unconjugated Synomag is less than 1 h [34] and we found the presence of both SI and SH nanoparticles 24 h after injection. This longer blood clearance time might allow them to interact with cells of the TME more than SP, thereby enhancing their accumulation in the TME, while most SP nanoparticles were engulfed predominantly by the liver Kupffer cells and splenic macrophages.

This highlights a gap in the current understanding of tumor targeting using antibody‐conjugated nanoparticles. Furthermore, we recently showed that pegylated Synomag can accumulate in the TME and enable detection of both primary and metastatic tumors by MPI. We did not see a difference between pegylated Synomag used in the previous study and an antibody‐conjugated Synomag nanoparticle type used in this study in terms of total MPI signal. We note that the pattern of nanoparticle accumulation (high at the edge and decreasing toward the center) seems specific to tumor‐associated inflammation, and that this is distinguishable in MPI from other inflammation. This bears further study, but because inflammation drives, sustains, and supports tumor growth in the host [35], it is attractive to surmise that patterns of nanoparticle accumulation may depend on the type of inflammation. This notion is intriguing and represents a potential opportunity to use iron oxide nanoparticle uptake and retention for detection by non‐invasive MPI. Future detailed studies are needed to test the limits of such a distinction. Because a tumor is often associated with a host‐mediated inflammatory response, MPI may be a complementary imaging modality to those already in use. MPI's specific attributes still need to be explored as it is still in its early stages of development. Recent clinical studies have shown that with the FDA‐approved iron oxide nanoparticle, Ferumoxytol, enhanced MRI can detect primary and metastatic lesions with the pattern of Ferumoxytol distribution showing enhancement around the periphery of the tumor tissue in patients [36, 37]. This presents further evidence that iron oxide nanoparticles can be used in the clinic for detection of both primary and metastatic tumors with MPI, which opens new avenues to explore the use of non‐radioactive, and non‐invasive MPI as an alternate approach.

## 3 Conclusions

In this study, we report observations that tumor specific antibody conjugated nanoparticles tend to be sequestered by phagocytic cells in the TME, rather than by cancer tumor cells. Indeed, we found no evidence of the latter. Therefore, both non‐specific and tumor specific antibody coated nanoparticles were engulfed in similar quantities by inflammatory cells residing in the vicinity of tumors, micrometastases, and organs of the reticuloendothelial system, in comparatively similar amounts. Unconjugated nanoparticles, however, were predominantly sequestered by the larger organs of the reticuloendothelial system. We saw substantial antibody‐conjugated nanoparticle accumulation in the TME that enabled non‐invasive detection of tumors by MPI. We also observed that MPI differentiated between tumor and wound‐associated inflammation because of differing patterns of nanoparticle distribution. These findings hold promise for developing iron oxide nanoparticle‐enabled MPI as a new modality for detecting tumors and metastases.

## 4 Methods/Experimental

### 4.1 Cell Lines and Reagents

Human Epidermal growth factor Receptor 2 (HER2) negative MDA‐MB‐231 and HER2 positive SKBR3 cells were purchased from American Type Culture Collection (ATCC) (Manassas, VA). Both cell types were grown in Dulbecco's Modified Eagle Medium (DMEM) (Thermo Fisher Scientific Inc., Waltham, MA) containing 10% Fetal Bovine Serum (FBS) (MilliporeSigma, Burlington, MA). All cell lines were authenticated using short tandem repeat (STR) analysis (data provided upon request) and matched against ATCC and Deutsche Sammlung von Mikroorganismen und Zellkulturen (DSMZ) databases to ensure their genetic origins. All other reagents used were of analytical grade.

### 4.2 Nanoparticle Synthesis, Conjugation, and Characterization

Synomag (hydroxyethyl starch coated) plain (SP), Synomag‐IgG (SI) and Synomag‐Her2 (SH) nanoparticles were obtained from Micromod Partikeltechnologie, GmbH (Rostock, Germany). Details of synthesis and conjugation is given below.

The Synomag plain (SP) nanoparticles were synthesized by a core‐shell method as described before [38, 39]. SPs with human IgG on the surface were prepared by maleimide functionalization of amino functionalized SP nanoparticles (105‐01‐102, micromod Partikeltechnologie GmbH) [40]. In a second step, the human IgG (Catalog #: 1‐001‐A, R&D Systems, Inc. Minneapolis, MN) was thiolated with Traut's reagent and conjugated with the maleimide functionalized particles [41] to get Synomag IgG (SI). SPs with Her2 antibody on the surface were prepared in an analogous way as were SI. Instead of human IgG, Her2 antibody (Catalog #: MAB9589, R&D Systems, Inc. Minneapolis, MN) was thiolated with Traut's reagent and reacted with the maleimide functionalized SPs to get Synomag HER (SH) [42].

The hydrodynamic diameter, size distribution, and zeta potential of each nanoparticle type were measured with a Zetasizer Ultra (Malvern Panalytical). Data are shown in Table S1. Particle size was measured at an iron concentration of 0.1 mg/mL in 0.22 µm filtered water. The zeta potential was measured at an iron concentration of 0.5 mg/mL in 1 mM KCl. The antibody concentration of SI and SH was measured as described before [19]. For visualization of nanoparticles, transmission electron microscopy (TEM) was used. Briefly, nanoparticles (8 µL) were adsorbed to glow discharged (EMS GloQube) ultra‐thin (UL) carbon coated 400 mesh copper grids (EMS CF400‐Cu‐UL), by floating for 1 min. Following aspiration, grids were imaged on a Hitachi 7600 TEM operating at 80 kV with an AMT XR80 CCD (8 megapixel).

### 4.3 Characterization of Antibody‐Specific Nanoparticle Uptake In Vitro by Ferene Assay and Prussian Blue Staining

MDA‐MB‐231 (HER2^−^) and SKBR3 (HER2^+^) cells were used to test the antibody‐specific uptake of Herceptin conjugated nanoparticles. For this, 1 × 10^6^ cells in 1 mL media (with 10% FBS) were incubated in a 37°C incubator with SP, SI or SH nanoparticles (0.5 mg Fe/mL) for 3 h in sterile polystyrene tubes. After incubation, tubes were centrifuged at 1,200 rpm for 6 min to aspirate and discard media. Cells were then washed with 2 mL of PBS and centrifuged as above. This was repeated three more times, and the final cell pellet was suspended in 1 mL of PBS. Cell number was counted using a Cellometer (Nexcelom Bioscience, Lawrence, MA) and a known number of cells was processed by ferene‐s assay to quantitate total iron inside cells as described before [19]. For the pre‐incubation study, SH nanoparticles (0.5 mg/mL) were pre‐incubated with media containing 10% FBS for 4 h at 37°C to allow serum proteins to form a protein corona around the nanoparticles. After incubation, this solution was added to SKBR3 cells (1 × 10^6^ cells). To compare the difference in uptake, with and without pre‐incubation another set of SKBR3 cells was incubated with nanoparticles (no pre‐incubation) and used for ferene assay after 3 h incubation as described above. A small quantity of cells (1 × 10^4^) was seeded in chamber slides (Thermo Fisher Scientific, Rochester, NY) and left overnight for attachment. The next morning, media was discarded and chambers were washed with PBS. Cells were then fixed with 10% formalin for 30 min at room temperature. After discarding formalin, chambers were washed with PBS and cells were stained with 20% hydrochloric acid and 10% potassium ferrocyanide solution (1:1 mixture made fresh) for 20 min to detect ferric ions. After washing, chambers were counterstained for 5 min with Nuclear Fast Red (Abcam, Cambridge, UK). After thorough washing in water, chamber slides were dried before mounting with mounting media. Cells were visualized and photographed using an EVOS M7000 microscope at 40x magnification (Thermo Fisher Scientific, Waltham, MA).

### 4.4 Iron Uptake by Macrophages

Iron uptake by cultured macrophages of distinct phenotype was assayed as described before [19]. Briefly, RAW264.7 cells (ATCC, Manassas, VA) maintained in DMEM with 10% heat‐inactivated FBS were treated with LPS (100 ng/mL; Sigma‐Aldrich, St. Louis, MO) and IFN‐γ (50 ng/mL; Miltenyi Biotech, Germany) for 24 h to get M1 macrophage phenotype. To induce cells into M2 phenotype they were treated with IL‐4 (10 ng/mL; Miltenyi Biotech, Germany) for 24 h [43]. Cells grown in complete media alone served as uninduced M0 phenotype. Induced and uninduced cells (1 × 10^6^) were then incubated with SP, SI or SH nanoparticles (0.5 mg/mL) for 24 h in their respective media. After incubation, cells were washed thoroughly with PBS four times and processed for intracellular iron content analysis by ferene‐s assay as described before [44]. All experiments were repeated four times.

### 4.5 Mouse Models

All animal studies were performed in strict accordance with the NIH guidelines for the care and use of laboratory animals [45]. The Institutional Animal Care and Use Committee at Johns Hopkins University approved all animal studies for investigator RI, protocol number MO24M16. We maintain a colony of transgenic Human HER2 overexpressing repetitive mice (HuHER2) on FVB/NJ background that spontaneously develop metastasizing (to lung) mammary tumors [19]. These mice were originally obtained from Genentech (San Francisco, CA) under a Materials Transfer Agreement. FVB/NJ were purchased from Jackson Labs, Bar Harbor, ME. All mice were fed a normal diet and water ad libitum. They were maintained in the normal 12 h of light and dark. All animals were monitored closely for any distress or pain throughout the study period.

### 4.6 In Vivo Study Design

HuHER2 transgenic mice with tumors: Transgenic mice develop single or multiple palpable mammary tumors when individuals are around 7–8 months of age. We monitored and measured all mammary tumors until they were 1600–1800 mm^3^ (total volume). We have previously shown that 42% of these mice will present with lung metastasis [46]. Mice bearing tumors were randomly assigned to one of four groups (*n* = 4–8/group) to receive a single intravenous injection of SP, SI or SH (1 mgFe/mouse, or 30 mg/kg b.wt) 72 h before MPI.

HuHER2 transgenic mice without tumors: Age‐matched HuHER2 transgenic mice having no palpable tumors were used as controls. These mice were injected with SI or SH (1 mgFe/mouse – n = 3/group) 72 h before MPI.

FVB/NJ wild type normal mice: Young female FVB/NJ mice (8‐10 weeks old) were used for no tumor control cohorts. We injected SI or SH (1 mgFe/mouse – *n* = 3/group) into these mice 72 h before MPI.

HuHER2 allograft model: HuHER2 allograft model developed as described before in 8 week‐old wildtype FVB/NJ mice [19]. When the tumor volume reached 150 – 200 mm^3^, mice were randomly assigned to receive an intravenous injection of PBS, SI or SH (0.5 mg or 1mgFe/mouse – *n* = 3/group). Twenty four hour after injection, all mice were sacrificed to collect tumors and organs to perform 2D MPI followed by dissection of organs. One half of each organ was fixed in 10% formalin for histological analysis by H&E, Prussian blue, and IHC; and the other half was used for ICP‐MS.

### 4.7 In Vivo Magnetic Particle Imaging and Micro‐Computed Tomography

MPI scans were performed using the same methods described previously [15]. In brief, we used a Momentum scanner for all MPI imaging in this work (Magnetic Insight, Alameda, CA). The mice underwent imaging under anesthesia 72 h post nanoparticle injection, while we monitored their respiration and internal body temperature (Small Animal Instruments, Stony Brook, NY). We first performed a 3D isotropic (*x* and *z* scan) in “Standard” mode with the magnet having a gradient strength of 5.7 T/m and an excitation field strength of 5 mT. Micro‐computerized tomography (µCT) (Perkin‐Elmer, Shelton, CT, USA) was then immediately used to gather anatomical imaging data. Small aliquots of iron oxide nanoparticles were used to co‐register the MPI and µCT images. Maximum intensity projections (MIPs) were created from each scan for qualitative analysis [22, 23]. For quantitative analysis, we segmented a small (∼35 mm^3^) portion of the tumor and lower left leg, where the average MPI signal from each tumor segmentation was compared against the corresponding signal from the muscle.

### 4.8 Euthanasia

After live imaging with MPI and µCT, all mice were euthanized to collect blood (in EDTA coated tubes) and tissues of interest. This includes tumors, lung, liver, spleen, inguinal lymph nodes, sternum (for bone marrow), kidneys and intestine. In some cases, brain, bone tissue and other areas of bright spots detected by MPI were also collected. All tissues and 100 µL blood were then imaged in the scanner. After imaging, whole lung and a piece of tissue from all other organs were fixed in 10% formalin and processed for histology and IHC as described below.

### 4.9 Ex Vivo Magnetic Particle Imaging of Organs

Upon euthanizing the mice and removing the organs of interest for MPI scanning; we used the same scanning parameters, except for a 10 mT excitation field to amplify smaller signals. The scans of each organ consisted of a 6 cm x 6 cm scan area (241 pixels x 241 pixels) or 58,081 pixels per scan. The total summed MPI signal within the scanned area (i.e., sum of the signal from each pixel) is represented as MPI signal (arbitrary units—a.u).

### 4.10 Histology

Tissue fixed in 10% formalin for 48 h was embedded in paraffin blocks, sectioned, and stained with hematoxylin and Eosin (H&E). Prussian blue (PB) staining was performed for identifying iron nanoparticles. Charged unstained slides were used for IHC. HER2‐IHC was used to determine metastatic nodules in HuHER2 mice. Five serial sections (100 µm apart) were used for analysis.

IHC for immune and stromal cell markers was performed on HuHER2 allograft tumors collected from PBS‐treated mice from a previous study [19]. Archived tissue samples were used to determine their spatial distribution and correlate with that of nanoparticles from Prussian blue staining in this study.

### 4.11 Immunohistochemistry

Immunohistochemistry (IHC) for human HER2, mouse CD45 and F4/80 were performed on lung sections to identify HER2^+^ metastatic tumor nodules. All immune cell and macrophage staining, respectively, in the HuHER2 transgenic cohort was done as described before [15]. F4/80 staining was also conducted on lung sections from old HuHER2 transgenic mice without tumors and FVB/NJ wild type mice. For determining the patterns of distribution, HuHER2 allograft tumors were stained for endothelial cells using CD31 (1:40 – Dianova‐ DIA 310, Hamburg, Germany); tumor cells using HER2 (1:400; Cell Signaling Technology‐ 29D8 Danvers, MA); macrophages using F4/80 (1:2000 – Serotec, now Biorad MCA497, Hercules, CA); fibroblast cells using alpha‐ smooth muscle actin (a‐SMA ‐1:200 – Abcam ab5694, Waltham, MA); dendritic cells using CD11c (1:100 – Cell Signaling 97585S, Danvers, MA); collagen using Masson's trichrome; and nanoparticles by Prussian blue (performed at JHU core facility). Detailed IHC protocols using the aforementioned antibodies have been previously described [20].

### 4.12 Image Analysis

All slides were digitized using a NanoZoomer‐SQ Digital slide scanner (Hamamatsu Photonics, Shizuoka, Japan) and evaluated using Aperio Imagescope software (version 12.4.6, Leica Biosystems, Deer Park, IL), QuPath software v0.5.0 (GitHub, San Francisco, CA) and Image J (Fiji). Tumor area and F4/80^+^ cell numbers in lungs and spatial analysis of immune cells, stromal cells and nanoparticles was performed as described before [15, 20].

### 4.13 Inductively Coupled Plasma Mass Spectroscopy (ICP‐MS) for Total Iron Quantitation

To verify the presence of iron within the organs and the trends observed with MPI, we performed ICP‐MS analysis on the organs collected from the HuHER2 allograft cohort as described before [47]. Briefly, tissues were digested in 2 mL of optima‐grade nitric acid using a MARS5 Xpress microwave (CEM Corporation, Matthews, NC). A 20 µL aliquot of the digest was diluted to a final volume of 10 mL and nitric acid concentration of 2%. An internal reference standard (LGC Limited, Teddington, UK) was incorporated into each sample to detect instrument deviation and a separate sample containing Seronorm Trace Elements Whole Blood (Sero AS, Bliingstad, Norway) was used to verify iron quantitation accuracy. The samples were then analyzed with an Agilent 7500ce ICP‐MS (Agilent Technologies, Santa Clara, CA) to measure the total amount of iron present within the sample. Total signal counts were compared against an iron calibration standard (ISQC Standard 21, LGC Limited, Teddington, UK) for iron quantitation.

### 4.14 Statistical Analysis

All statistical analyses were performed using Graphpad Prism v6 software (Graphpad Inc, La Jolla, CA). Non‐parametric Mann‐Whitney tests were used for group comparisons. Data represent individual values with median, and *p* ≤ 0.05 was deemed significant. Both Spearman's correlation coefficient (*ρ*) and *p*‐value were used to determine the correlation between two groups.

## Author Contributions

Conceptualization: P.K., H.C., and R.I.; Methodology: P.K., H.C., T.S., T.W., S.H., M.S., P.W.G., and K.G.; Investigation: P.K., H.C., T.W., and M.S.; Visualization: P.K., H.C., and T.S.; Funding acquisition: R.I. and P.K.; Project administration: R.I. and P.K.; Supervision: R.I. and P.K.; Writing – original draft: P.K., H.C., and R.I.; Writing – review & editing: P.K., H.C., T.W., S.H., T.S., M.S., P.W.G., C.G., K.G., and R.I.

## Funding

This study was funded by the Jayne Koskinas Ted Giovanis (JKTG) Foundation for health and policy, a Maryland private foundation dedicated to effecting change in the health care industry for the greater public good (PK, RI), National Institutes of Health grant R01CA257557 (RI), National Institutes of Health grant R50CA305053 (PK). Magnetic Particle Imaging scanner core, ICP‐MS core, JHU Animal facility core, and other resource facilities were supported in part by National Institutes of Health grants S10OD026740, 1S10OD030355‐01, and P30CA006973. The contents are sole responsibility of the authors and do not represent the official view of the National Institutes of Health, JKTG Foundation or Johns Hopkins University. Funders had no role in the writing of the manuscript or in the decision to publish. This manuscript is the result of funding in part by the National Institutes of Health (NIH). It is subject to the NIH Public Access Policy. Through acceptance of this federal funding, NIH has been given the right to make this manuscript publicly available in PubMed Central upon the Official Date of Publication, as defined by NIH.

## Conflicts of Interest

T.S. and P.W.G. were employees at Magnetic Insight, Inc, C.A. and C.G. is an employee of micromod Partikeltechnologie, GmbH, Germany manufacturer of Synomag nanoparticles used in this study. R.I. is an inventor listed on several nanoparticle patents. All patents are assigned to Johns Hopkins University or Aduro Biosciences, Inc. All other authors declare they have no conflicts of interest.

## Supporting information




**Supporting File 1**: advs77425‐sup‐0001‐SuppMat.docx.

**Supporting File 2**: advs77425;sup‐sup;sup‐0002;sup‐S1.docx.

## Data Availability

The HuHER2 transgenic mouse model was procured from Genentech, Inc. through a Materials Transfer Agreement. All data needed to evaluate the conclusions in the paper are present in the paper, supplementary materials and all raw data files and original image files are accessible through  https://doi.org/10.7281/T1DADMSK, Johns Hopkins Research Data Repository, V1.
