# Supplementary material for: Hyper‐Intense Tumor Peripheral Accumulation of Antibody‐Conjugated Iron Oxide Nanoparticles can Enable Breast Cancer Detection by Magnetic Particle Imaging
Source: Adv Sci (Weinh). 2026 Aug 30:e77425. Online ahead of print. doi: 10.1002/advs.77425 (PMC13526418; doi:10.1002/advs.77425)
Supplement: Supplementary file 1 [file ADVS-9999-e77425-s001.docx]

**Figure S1**: A) SH nanoparticles with or without pre-incubation (4 h) in serum-containing media did not show any significant difference in uptake by HER2^+^ SKBR3 cells. B) Dose-dependent MPI signal detected from 2D ex-vivo scans of various organs. C) ICP-MS analysis quantifying absolute iron content in various organs also showed dose-dependent iron. D) A correlation analysis between iron quantified by MPI and ICPMS in organs shows a positive correlation with a Spearman correlation coefficient of 0.82.

**Figure S2: Spatial analysis for antibody-conjugated nanoparticle distribution showed a pattern of hyperintense peripheral accumulation.** A) Representative images showing nanoparticle distribution in the tumor by Prussian blue staining. Non-specific IgG (BI) or anti-HER2 antibody (BH) conjugated IONP showed intense peripheral Prussian blue positivity. B) Representative image of Prussian blue-stained tumor shows the workflow for pixel positivity analysis. C & D) PBS or unconjugated IONP (BP) treated tumors did not show a distinct pattern of distribution. Although a higher number of nanoparticles was detected in the BP cohorts compared to PBS controls, it was significantly lower than in the BI and BH cohorts. E &F) Both BI and BH had significantly higher peripheral accumulation of nanoparticles than their inner quartile.

**Figure S3:** Schema of the study

**Figure S4:** (A, B) Comparison of age and body weight of all HuHER2 transgenic mice showed no significant difference. (C, D) Likewise, measured tumor volume and wet weight after sacrifice were comparable between treatment cohorts. (E, F) Age and body weight of young and HuHER2 transgenic mice used in the study as a control cohort are shown (G) Quantitation of 2D MPI data showed significant differences between SP and SI/SH nanoparticle received cohorts, except in the blood in HuHER2 transgenic mice. While no significant difference was noted in non-tumor bearing mice (Mann Whitney - **p<0.01).

**Figure S5:** Images acquired from each SI-injected mouse using MPI, micro-computed tomography (µCT) of the lung area, 2D MPI of lung and its quantitation, measured lung tumor area and macrophage population in lung (F4/80 + cells) as described in the Methods. Primary tumors are indicated using an orange arrow. (WT – Normal non-tumored FVB/NJ wildtype mice, NT- non-tumored age-matched HuHER2 transgenic mice, T- tumored mice, # - indicates 3D measurement was not performed).

**Figure S6:** Images acquired from each SH-injected mouse using MPI and micro-computed tomography (µCT) of the lung area. 2D MPI of lung and its quantitation, measured lung tumor area and macrophage population in lung (F4/80 + cells), quantified as described in the methods, is given. Primary tumors are indicated using an orange arrow. (WT – Normal non-tumored FVB/NJ wildtype mice, NT- non-tumored age-matched HuHER2 transgenic mice, T- tumored mice, # - indicates 3D measurement was not performed).

**Figure S7:** Prussian blue staining shows accumulation of nanoparticles in all analyzed organs which corroborated with the 2D MPI analysis

**Figure S8:** SI injected mice shows nanoparticles (by Prussian blue staining) in immune rich CD45+ve and macrophage areas (F4/80+ve) than in HER2+ve tumor areas. Nanoparticles were seen in F4/80+ve areas even with absence of HER2+ve tumor.

**Figure S9:** As observed in SI injected mouse, SH injected ones also showed Prussian blue positive nanoparticles in immune rich CD45+ve and macrophage areas (F4/80+ve) than in HER2+ve tumor areas. Nanoparticles were seen in F4/80+ve areas even with absence of HER2+ve tumor.

**Figure S10: *In vitro* nanoparticle uptake measured by Ferene assay showed significantly higher uptake of IONPs by macrophages based on their phenotype**. When RAW264.7 macrophages were induced to M1 phenotype using LPS and IFN-γ, they took up significantly higher amounts of nanoparticles than when they were in naïve M0 condition of anti-inflammatory M2 (IL4 treated) state. A trend of higher SI and SH nanoparticle uptake was seen compared to SP nanoparticles in M1 induced macrophages. (Mann Whitney - *p<0.05)
