# Supplementary material for: Hyper‐Intense Tumor Peripheral Accumulation of Antibody‐Conjugated Iron Oxide Nanoparticles can Enable Breast Cancer Detection by Magnetic Particle Imaging
Source: Adv Sci (Weinh). 2026 Aug 30:e77425. Online ahead of print. doi: 10.1002/advs.77425 (PMC13526418; doi:10.1002/advs.77425)
Supplement: Supplementary file 2 [file ADVS-9999-e77425-s002.docx]

**Table S1: Analytical data of nanoparticles used in this study**

| **Particle type** | **Coating** | **Diameter [nm]** | **PDI** | **Lot. No:** | **ZP [mV]** | **Protein concentration [µg/mg Fe)** |
| --- | --- | --- | --- | --- | --- | --- |
| **Synomag-Plain (SP)** | Hydroxy ethyl starch | 106.7 | 0.064 | 30023105-01 | - 0.254 | - |
| **Synomag-IgG (SI)** | Human polyclonal IgG | 109.6 | 0.106 | 34823105-01 | + 6.363 | 34.4 |
| **Synomag-HER (SH)** | Herceptin | 127.4 | 0.115 | 35023105-01 | - 4.958 | 21.0 |
